# Domestic dogs as a comparative model for social neuroscience: Advances and challenges

**DOI:** 10.1016/j.neubiorev.2024.105700

**Published:** 2024-05-04

**Authors:** Magdalena Boch, Ludwig Huber, Claus Lamm

**Affiliations:** a Social, Cognitive and Affective Neuroscience Unit, Department of Cognition, Emotion, and Methods in Psychology, Faculty of Psychology, University of Vienna, Vienna 1010, Austria; b Department of Cognitive Biology, Faculty of Life Sciences, University of Vienna, Vienna 1090, Austria; c Comparative Cognition, Messerli Research Institute, University of Veterinary Medicine Vienna, Medical University of Vienna and University of Vienna, Vienna 1210, Austria; d Vienna Cognitive Science Hub, University of Vienna, Vienna 1010, Austria

**Keywords:** Comparative neuroimaging, Vision, Social cognition, Dog, Human

## Abstract

Dogs and humans have lived together for thousands of years and share many analogous socio-cognitive skills. Dog neuroimaging now provides insight into the neural bases of these shared social abilities. Here, we summarize key findings from dog fMRI identifying neocortical brain areas implicated in visual social cognition, such as face, body, and emotion perception, as well as action observation in dogs. These findings provide converging evidence that the temporal cortex plays a significant role in visual social cognition in dogs. We further briefly review investigations into the neural base of the dog-human relationship, mainly involving limbic brain regions. We then discuss current challenges in the field, such as statistical power and lack of common template spaces, and how to overcome them. Finally, we argue that the foundation has now been built to investigate and compare the neural bases of more complex socio-cognitive phenomena shared by dogs and humans. This will strengthen and expand the role of the domestic dog as a powerful comparative model species and provide novel insights into the evolutionary roots of social cognition.

## 1 Introduction

We frequently encounter and engage with others in our daily lives, providing us with a wealth of social cues. These cues, including emotional expressions, gestures, and ostensive communicative signals, are vital in helping us navigate our intricate social world. They assist us in avoiding potential harm, guiding our social interactions, fostering relationships, and facilitating integration within social groups. The ability to perceive, integrate, and interpret the social contextual information surrounding us is often considered what makes humans stand out compared to other species (e.g., Frith, 2008 for review). However, much remains to be uncovered about the evolutionary origins of these advanced socio-cognitive skills.

Comparing the structure and function of brains across species offers a unique opportunity to investigate the evolutionary history of social cognition (Roberts et al., 2022). Decades of comparative neuroimaging research with non-human primates have provided new insights into the evolutionary roots of human social cognition (Friedrich et al., 2021; Rilling, 2014). However, human social behaviour evolved by adapting to changes in their complex social environment (Barsbai et al., 2021; Dunbar and Shultz, 2007); this is difficult to study by comparative research between humans and non-human primates because they exhibit many differences beyond social behaviour, such as language or tool-use. An additional, complementary approach is to look for convergent evolution in a different lineage. Convergent evolution describes when two (distant) species evolved, for example, a similar (i.e., analogous) ability or trait which was not present in their last common ancestor and resulted from shared evolutionary pressures (Roberts et al., 2022).

Over the last two decades, dogs (*Canis familiaris*) have emerged as an exciting model species to study the evolutionary basis of social abilities and a potential convergence with those of humans. As close companions for millennia (Bergström et al., 2020), the two species have shared the same ecological niche and social environment, and dogs have been domesticated by humans (Range and Marshall-Pescini, 2022). A growing body of evidence has identified numerous analogous behavioural correlates of human visual socio-cognitive skills in dogs and humans (Huber, 2016; Kujala, 2017). For example, dogs have excellent face perception skills; they can, e.g., discriminate between positive and negative emotional expressions (Albuquerque et al., 2016; Müller et al., 2015; Nagasawa et al., 2011) and detect familiarity (Huber et al., 2013; Pitteri et al., 2014; Racca et al., 2010). Dogs are also sensitive to bodily-referential and ostensive cues, such as pointing gestures or eye-gaze (Bray et al., 2021; Duranton et al., 2017; Kaminski et al., 2013; Soproni et al., 2002; Téglás et al., 2012; Topál et al., 2014), and they tend to imitate actions of others, similar to humans. Unlike rhesus macaques, dogs can match actions in a “Do-as-I-do” training paradigm (Fugazza et al., 2019; Fugazza and Miklósi, 2014; Topál et al., 2006), they spontaneously match human actions already as puppies (Fugazza et al., 2023), and even over-imitate action sequences demonstrated by their human caregivers (Huber et al., 2018, 2020). Furthermore, dogs also share more complex socio-cognitive abilities with humans: they can perform visual perspective taking (Catala et al., 2017; Maginnity and Grace, 2014; and see Huber and Lonardo, 2023 for review), respond to unfair treatment (i.e., inequity aversion; (Brucks et al., 2016; Essler et al., 2017; Range et al., 2009; McGetrick and Range, 2018), form expectations about physical events (Völter, Tomašić, et al., 2023; Völter and Huber, 2021a, 2021b) and are sensitive to humans’ intentions, knowledge or beliefs (Lonardo et al., 2021; Schünemann et al., 2021; Virányi et al., 2006; Völter, Lonardo, et al., 2023). Finally, dogs and humans also display relevant differences in their abilities and behaviours, apart from unique human abilities such as language. Dogs are, for example, not known as tool users and primarily manipulate or explore objects with their snouts. Thus, dogs constitute an exciting model species to probe the evolutionary roots of social cognition and behaviour.

Recent advances in non-invasive functional magnetic resonance imaging (fMRI) with dogs now allow researchers to expand from studying behavioural to neural correlates of putatively convergent and divergent visual socio-cognitive abilities. Unlike other comparative animal models, dogs can be specifically trained to participate in fMRI studies fully awake and without any restrain (Berns and Cook, 2016; Karl, Boch, Virányi, et al., 2020; Strassberg et al., 2019), and the majority of the studies are conducted with pet dogs living in human households. This allows for comparative studies with experimental set-ups largely identical to those used for human participants. Neuroimaging studies with pet dogs in the last decade have uncovered first functional analogies in the dog and human brains during visual social information processing. Here, we review what we have learned so far about how the dog brain processes visual social cues with a focus on neocortical areas and discuss functional similarities and differences with the human brain. Given that dogs are still a relatively novel model species in comparative neuroimaging (Thompkins et al., 2016), we start by providing a brief overview of the neocortical organization of the dog in comparison to the human brain and the resulting implications for interpreting the results. We then summarize the findings so far, which we roughly divided into three thematic categories: face, body and emotion perception, action observation, and neural bases of the dog-human relationship. We then discuss two main challenges in the field and provide suggestions on how to overcome them. Finally, we discuss future directions of the field and argue that findings so far provide the foundations to start investigating the neural bases of more complex visual socio-cognitive skills, such as empathy and theory of mind.

## 2 Main text

### 2.1 Neocortical organization of the dog and human brain

**Fig. 1:**
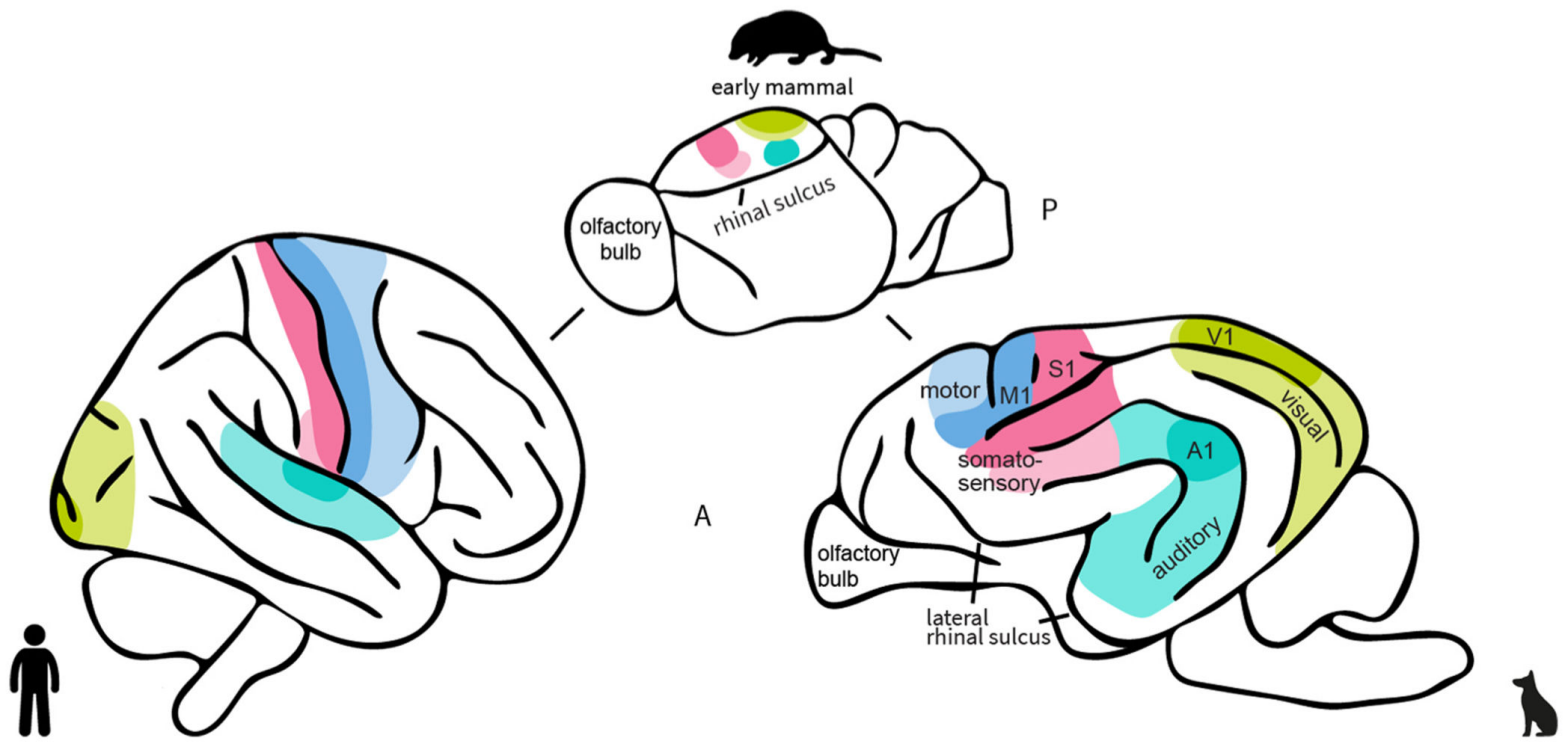
Illustration of the dog and human neocortex (lateral view) and how it evolved from their last common ancestor. The primate and carnivoran lineage split over 95 million years ago. The neocortex of both species vastly expanded after they diverged, and neocortical structures such as the temporal lobe evolved independently of each other in the two lineages (Kaas, 2013; Lyras, 2009). Therefore, brain regions beyond primary sensory areas should not be considered homologous. V1, primary visual cortex; A1, primary auditory cortex; A, anterior; P, posterior. The drawing of the early mammalian brain is based on Kaas (2013).

First and foremost, it is important to note that, although tempting, assumptions of homologies between dog and human brains should not be made based on relative location in the brain - such as, for instance, that dog ventral temporal areas responsive to faces must be homologues of the ventral human fusiform face area. This would be misleading as the last common ancestor of dogs and humans lived approximately 95–100 million years ago and had a smooth brain with a significantly larger allo-than neocortex, which mainly consisted of unimodal primary and sensory cortical areas (Kaas, 2011, 2013; Krubitzer et al., 2011; and **see** Fig. 1). Thus, the neocortex of primates and carnivorans largely expanded after they split, and especially higher-order unimodal and multimodal sensory regions should, therefore, not automatically be considered homologous. Primary sensory areas are considered homologous, as they have been observed in many living mammalian species, and they exhibit similar relative positions, such as the primary visual cortex (V1) housed in the posterior part of the brain. However, as pointed out by Krubitzer et al. (2011) in the example of rodent species, the organisation of primary sensory areas also varies across species because they continue to evolve and adjust to the species’ ecology and behaviour. Overall, the concept of homology based on morphology is not easily applicable to the study of brain organisation, especially on the level of brain function as compared to structure, and there is no uniformly accepted theory of homology for brain evolution (Sereno & Tootell, 2005; Strausfeld & Hirth, 2013; Striedter, 2002).

In the following section, we will provide a brief overview of dog in relation to human neuroanatomy and knowledge so far about approximate locations of sensory areas in the dog brain, mainly derived from available electrophysiological and histological work in domestic dogs and African wild dogs, their evolutionary relatives. This will serve as a first roadmap to navigate the dog brain before we continue to review insights gained from comparative neuroimaging on potentially shared neural correlates of visual social perception in dogs and humans.

### 2.2 Neuroanatomy of the dog brain and knowledge about sensory areas

**Fig. 2:**
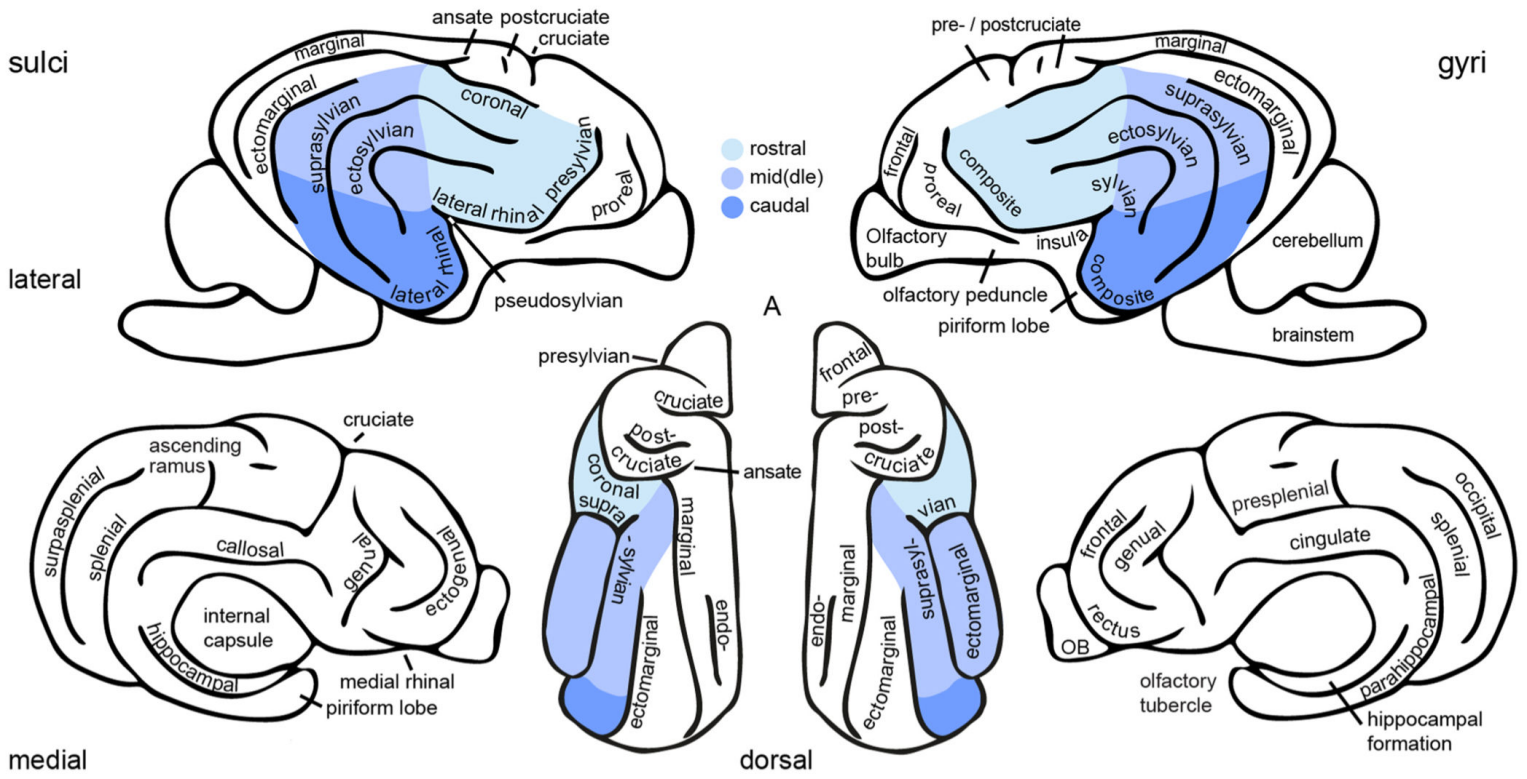
Sulci and gyri of the dog brain. The schematic illustration shows the main sulci and gyri of the dog brain accompanied by selected sub-cortical anatomical landmarks to facilitate visual guidance. The left side of the figure illustrates the sulci, the right side the gyri, each displayed from lateral, medial and dorsal view. Nomenclature follows the detailed description of (Fletcher and Beitz, 2013) and (Czeibert et al., 2018). A, anterior; OB, olfactory bulb.

**Fig. 3:**
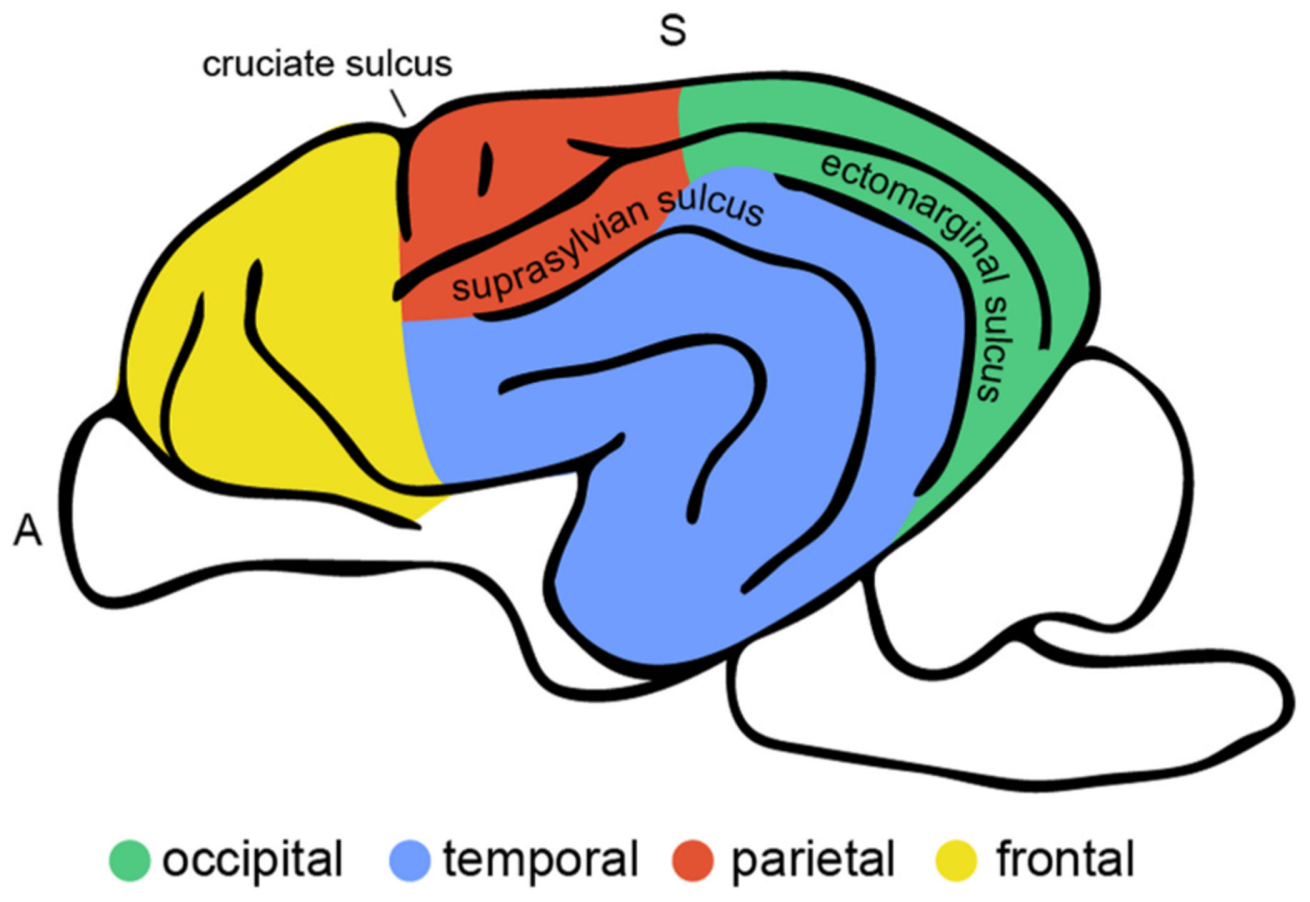
Illustration of (tentative) dog brain lobe assignments in the present review. There is no common agreement on the exact borders of each lobe in the dog brain. Here, we illustrate our categorisation of dog brain lobes based on existing labels (see, e.g., Garin et al., 2022; Johnson et al., 2020; Nitzsche et al., 2019), complemented by novel observations of brain function in dogs, as outlined in the present review. The ectomarginal sulcus constitutes the border between the occipital and temporal lobe, and the ascending ramus (medial wall) marks the transition from the occipital to the parietal lobe. The cruciate sulcus is the border between the parietal and frontal lobe and the rostral suprasylvian sulcus is between the parietal and temporal lobe.

One of the many differences between the dog and human brain is the shape of the temporal lobe, which evolved independently in the two species (Bryant and Preuss, 2018; Fletcher and Beitz, 2013; Uemura, 2015). The temporal lobe is a structure that is not present in all mammalian brains; it is thought that the primate temporal lobe underwent an independent evolution from the temporal brain extensions seen in carnivorans and cetaceans (Bryant and Preuss, 2018). In dogs, unlike in humans, the (pseudo-)sylvian sulcus or fissure does not constitute the border to the frontal and parietal lobe but the centre of the temporal lobe with the temporal (or perisylvian) gyri folded around the sulcus (see Figs. 2–3). Prior histological and electrophysiological research indicates that similar to humans, the temporal lobe of dogs, houses auditory and visual (Adrian, 1941; Pinto Hamuy et al., 1956; Tunturi, 1944), but also multisensory association regions located in the rostral and mid sylvian and caudal composite gyrus (Kosmal, 2000; Kosmal et al., 2004; see Fig. 1 for illustration). This has also been shown in functional MRI studies in dogs (Aguirre et al., 2007; Andics et al., 2014, 2016; Boch et al., 2021; Guran et al., 2024); and histological research in African wild dogs, evolutionary relatives of domestic dogs, also show that the canine temporal lobe houses visual and auditory regions (Chengetanai et al., 2020). While there is common agreement that the sylvian, ectosylvian and caudal composite gyri are part of the temporal lobe, definitions vary whether the mid and caudal suprasylvian gyrus is considered occipital or temporal (Johnson et al., 2020; Nitzsche et al., 2019). In the present review, we define the mid and caudal suprasylvian gyrus as part of the dog temporal lobe due to accumulating evidence for its *functional* convergence with the human inferior temporal lobe, outlined in detail in the following section.

The occipital lobe of dogs houses the visual cortex, but other than in humans, the dog’s primary visual cortex (V1) is located at the posterior portion of the dorsal marginal gyrus and not at the occipital pole (see Fig. 1), as indicated by electrophysiological work in dogs (Fletcher and Beitz, 2013; Ofri et al., 1995), and histological research in African wild dogs (Chengetanai et al., 2020).

Histological research in African wild dogs suggests the visual cortex further expands to the parietal lobe (Chengetanai et al., 2020; i.e., the anterior ectomarginal, marginal, and presplenial gyrus), and these areas have been implicated in processing visual numerical information using task-based fMRI (Aulet et al., 2019). However, more research is needed to determine if this region of the parietal cortex of dogs exclusively processes visual information. Electrophysiological research in domestic dogs (Pinto Hamuy et al., 1956) shows that the rostral suprasylvian gyrus and posterior portions of the postcruciate gyrus house the primary somatosensory cortex (S1; see Fig. 1). The rostral suprasylvian gyrus is mainly involved in processing sensory information from the head; the ventral postcruciate gyrus processes sensory information received from the forepaws- and arms and then continues to process posterior body parts moving further dorsally, with sensory information from the tail being processed on the medial wall (Adrian, 1941; Pinto Hamuy et al.,1956). A similar somatotopic organisation has also been observed in other Carnivoran species, with a more expanded postcruciate gyrus in species primarily using their forepaws and -arms, such as the red panda, raccoon or coati (Welker and Campos, 1963; Welker and Seidenstein, 1959). S1 was recently also successfully located using a non-invasive functional MRI localiser with awake dogs (Guran et al., 2024).

Anterior to the postcruciate sulcus, the postcruciate gyrus houses the primary motor cortex, which curves ventrally around the cruciate sulcus, bordering the premotor and supplementary motor cortex in the precruciate gyrus, as indicated by histological research (Stanton et al., 1986; Tanaka et al., 1981). Thus, as in humans, the motor and somatosensory regions are housed adjacently, but in humans, the border between S1 and M1 is marked with a more pronounced sulcus (i.e., central sulcus; see Fig. 1).

The functional properties of frontal lobe regions remain largely unstudied beyond the premotor cortex and are also difficult to investigate with functional MRI due to the dogs’ large air-filled nasal cavities affecting the signal in this area (see e.g., Boch, Karl, et al., 2023; Szabó et al., 2019). Research on sulcal evolution in Canidae, however, indicates an expansion of the proreal gyrus with the evolution of pack structures in more social compared to primarily solitary living canine species (Radinsky, 1969).

Another intriguing point of comparison lies in the varying degrees of expansion of the lobes across the two species. While the human brain has significantly more expanded frontal and parietal than temporal and occipital lobes, the opposite pattern is observed in dogs (Garin et al., 2022), as can be seen by the significant expansion of their occipital and temporal lobes in Fig. 3. Thus, dog and human brain structural macro-anatomical organization differs substantially in some aspects. However, the two brains also share many similarities, making it highly interesting to investigate how dogs’ and humans’ analogous socio-cognitive skills come about in the two brains, and especially whether functionally analogous neural correlates can be found.

### 2.3 Dog fMRI research so far: insights into the neural bases of attachment, emotion, agent and action perception using visual social cues

**Fig. 4:**
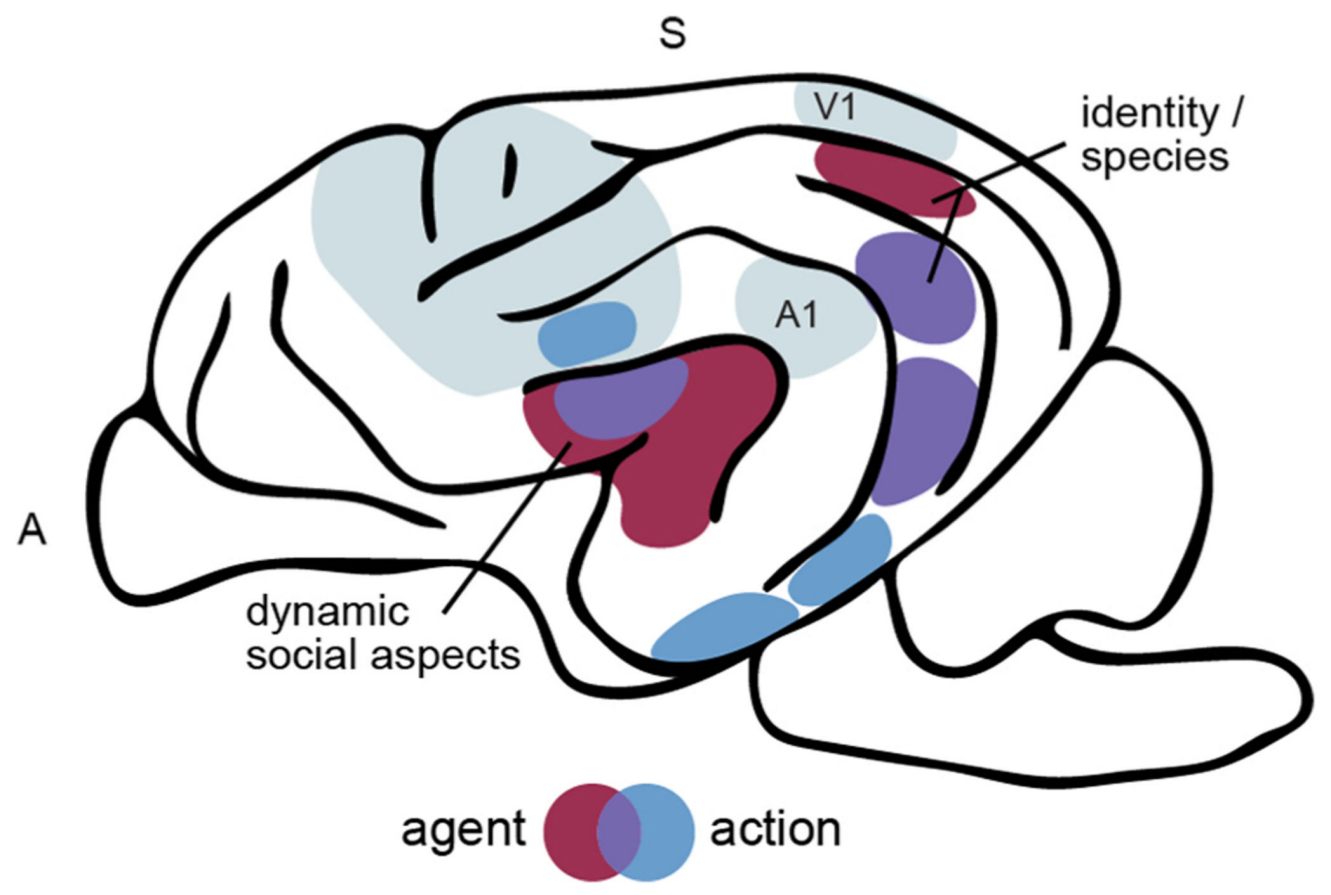
Converging evidence for a predominant role of the temporal lobe for visual social perception in dogs. Functional MRI studies so far localized neocortical areas involved in agent (i.e., faces and bodies; (Boch, Wagner, et al., 2023; Dilks et al., 2015; Szabó et al., 2020) and action (Phillips et al., 2022) perception and showed that these areas exchange information (i.e., task-based functional connectivity) with primary visual cortex (V1) during face, body and action perception (Boch, Karl, et al., 2023). First evidence suggests that areas in the multisensory sylvian gyrus process dynamic social aspects of visual social cues (e.g., emotion perception or social interactions; (Boch, Karl, et al., 2023; Hernández-Pérez et al., 2018; Karl et al., 2021; Phillips et al., 2022) and sensitivity for species identity in the ectomarginal and mid suprasylvian gyrus (Boch, Karl, et al., 2023; Bunford et al., 2020). However, investigations into the specific roles of the localized agent and action areas remain to be undertaken. The figure represents a schematic summary of the findings with approximate locations of activated areas; please refer to the text and Supplementary Table S1 for more detailed information. If multiple neuroimaging studies investigated the same research question (e.g., functional specialization for faces), areas where the majority of the studies converge are indicated. A, anterior; S, superior; A1, primary auditory cortex.

Over the last decade, six different labs have conducted a total of 16 dog fMRI studies investigating how dogs perceive visual social cues with topics spanning from (1) face, body and emotion perception to the neural underpinnings of (2) action observation and (3) the dog-human relationship (see Table S1 for study overview and Fig. 4 for a summary of the main findings).

#### 2.3.1 Face, body and emotion perception

The majority of the fMRI studies exploring the neural bases of agent perception focused specifically on face processing (Bunford et al., 2020; Cuaya et al., 2016; Dilks et al., 2015; Gillette et al., 2022; Hernández-Pérez et al., 2018; Szabó et al., 2020; Thompkins et al., 2018) with two studies, additionally investigating body perception (Boch, Wagner, et al., 2023). All studies show converging evidence that the occipito-temporal lobe plays a key role in agent perception in dogs, which is in line with similar findings in humans, suggesting a possible analogy. The brain areas where most of the studies converge are the ectomarginal (extrastriate cortex) and the temporal mid and caudal suprasylvian and sylvian gyrus (see Fig. 4 for a schematic summary of the core agent-responsive areas).

Studies so far showed that these agent-responsive areas result in greater activation for faces compared to inanimate objects (Boch, Wagner, et al., 2023; Cuaya et al., 2016; Dilks et al., 2015; Gillette et al., 2022) and scenes (Dilks et al., 2015), but not all found greater activation to scrambled controls (Boch, Wagner, et al., 2023; Dilks et al., 2015; Szabó et al., 2020). The ectomarginal, mid and caudal suprasylvian agent gyrus were also more active during perception of bodies than inanimate objects and scrambled controls (Boch, Wagner, et al., 2023) but seem to be largely involved in the perception of both faces and bodies, except for a patch in the suprasylvian gyrus, which resulted in greater activation for bodies compared to faces. Viewing bodies compared to faces or inanimate and scrambled controls also resulted in greater task-based functional connectivity between the primary visual cortex (V1) and the caudal suprasylvian agent-responsive area (Boch, Karl, et al., 2023). Task-based functional connectivity measures between the mid suprasylvian agent-responsive area and V1 did not differ between face and body perception but were significantly higher compared to the control conditions. This aligns with the activation-based analyses and indicates that face-sensitive areas also respond to observing other body parts. Overall, (limited) findings so far suggest a functional specialization for body perception analogous to humans (Downing et al., 2006), but whether dog agent-responsive areas also house patches specialized for face perception analogous to humans, such as the fusiform face area (FFA) remains debatable ((Burns et al., 2019).

Except for one study (Thompkins et al., 2018), which suggests the presence of separate areas for processing of human and conspecific (i.e., dog) faces, evidence so far suggests that the dog agent-responsive areas are involved both in the perception of human and conspecific faces and bodies (Cuaya et al., 2016; Dilks et al., 2015; Gillette et al., 2022), but that the mid suprasylvian gyrus responds stronger to the viewing of conspecific agents (Bunford et al., 2020). Similar observations have also been made in non-human primates and less pronounced in humans (Blonder et al., 2004; Boch, Wagner, et al., 2023; Bunford et al., 2020; Hori et al., 2021; Tsao et al., 2003). Considering the strong bond and co-habitation with humans, future research should also incorporate images of facial and bodily stimuli of other animals to address how dog agent-responsive areas respond to heterospecific agents and if these areas respond more generally to the presentation of faces and bodies.

The sylvian gyrus agent-responsive area was especially sensitive to the live and direct viewing of faces (Gillette et al., 2022; Szabó et al., 2020) and was, together with limbic regions such as the caudate or amygdala, more active when dogs viewed images of happy compared to neutral human faces (Hernández-Pérez et al., 2018; Thompkins et al., 2021). Using multivariate pattern analysis (MVPA; Hernández-Pérez et al., 2018) further demonstrated that happy human faces elicited a distinct neural activation pattern in the sylvian gyrus, which can be differentiated from neural representations of other basic emotions. Thus, the sylvian gyrus might be especially sensitive to dynamic aspects of visual social cues, analogous to the human lateral temporal pathway areas such as the posterior superior temporal sulcus (e.g., Yang et al., 2015 or Pitcher and Ungerleider, 2021; Wurm and Caramazza, 2022). The ectomarginal and suprasylvian agent-responsive areas, on the other hand, appear partly functionally analogous to the human extrastriate and inferior temporal agent-responsive areas (Mur et al., 2013; Schwarzlose et al., 2005; or see Kanwisher, 2010 for review), except for the potential lack of specialization for faces.

#### 2.3.2 Action perception

Compared to face and body perception, action perception has been studied less, with only three recent studies (Boch, Karl, et al., 2023; Karl et al., 2021; Phillips et al., 2022) investigating various aspects of action observation. The results suggest a predominant role of the temporal lobe, especially in the caudal composite, rostral sylvian and ectosylvian gyrus (see Fig. 4 for a schematic overview).

Boch, Karl et al. (2023) found significantly stronger temporal than parietal lobe involvement in dogs, contrary to humans, during action observation. This was derived from the extent of univariate activation and the strength of task-based functional connectivity with early visual cortex. Observing a dog or human picking up a visible (transitive) or invisible (intransitive) toy compared to visual and motion controls led to activation in the temporal mid and caudal suprasylvian agent-responsive areas, which were identified using an independent functional localizer, and additionally in the caudal composite and rostral sylvian gyrus, and somatosensory regions. The study also found overall greater task-based functional connectivity between V1 and the temporal than parietal lobe and the strongest V1 connectivity during action observation compared to the controls in the temporal action- and agent-responsive areas. Parietal cortex activation was significantly less pronounced in dogs than in humans during action observation, with action-responsive areas beyond the somatosensory cortex only localized in the human parietal lobe.

Regarding the perception of different types of actions, observation of transitive and intransitive actions elicited the same action- and agent-responsive areas with no pronounced differences in activation both in dogs and humans (Boch, Karl, et al., 2023). Moreover, (Karl et al., 2021) found stronger engagement of the mid suprasylvian, rostral sylvian and ectosylvian (i.e., secondary somatosensory cortex) agent- and action-responsive areas together with limbic structures during the observation of social compared to non-social interactions (see also next section for further discussion of this study). Employing a machine learning approach, (Phillips et al., 2022) further showed that a classifier could successfully be trained to discriminate multivariate activation patterns for viewing different types of actions (e.g., sniffing, eating or playing) performed by dogs, humans or other non-human animals (such as cats, deers or squirrels). However, only in humans the classifier also performed well in discriminating activation patterns for agent and inanimate object observation. Regions carrying important information for the action-classifier were widespread in the dog brain but again included temporal suprasylvian and rostral sylvian gyrus areas, and somatosensory regions (i.e., rostral ectosylvian gyrus). Most informative human brain regions were mainly located in the posterior temporal lobe (i.e., the area centred on pSTS).

In sum, the findings further emphasise the involvement of the dog sylvian gyrus during observing dynamic aspects of visual social cues and its role in the integration of social information, which could be considered analogous to the engagement of the human lateral temporal pathway (Pitcher and Ungerleider, 2021; Wurm and Caramazza, 2022). The mid suprasylvian agent-responsive area again responded stronger to conspecifics than to humans (Boch, Karl, et al., 2023), but overall, the three available studies suggest that the dog action observation network is engaged similarly during the perception of actions performed by conspecifics, humans, and other non-human animal agents (Boch, Karl, et al., 2023; Karl et al., 2021; Phillips et al., 2022). Importantly, action observation resulted in strong temporal lobe engagement (Boch, Karl, et al., 2023; Karl et al., 2021), which can be considered functionally analogous to observations in common marmosets (Zanini et al., 2023) but differs to humans, who show a more distributed activation in parietal and temporal regions during action observation (Hecht et al., 2013). Unlike humans, apes, and Old World monkeys, dogs and common marmosets also have significantly more expanded occipital and temporal than frontal and parietal lobes (Garin et al., 2022), further emphasising a likely divergent evolution of the dog and human parietal lobe. One potential explanation for the observed differences might be grounded in the occurrence of complex object-manipulating behaviours in humans (Peeters et al., 2009; Orban and Caruana, 2014; Stout and Hecht, 2017 for reviews), but more comparative research is needed to investigate this hypothesis further and to determine the functions of the dog parietal lobe. Unfortunately, we cannot draw conclusions about frontal lobe involvement during action observation in dogs due to severe signal distortions in this area caused by the dogs’ large air-filled nasal cavities surrounding it (see e.g., Boch, Karl, et al., 2023 for discussion).

#### 2.3.3 Dog-human relationship

The special relationship between human caregivers and their dogs has been argued to resemble the attachment bond between human parents and their infants (Archer, 1997; Topál et al., 1998), which has motivated investigations into the neural bases underpinning the dog-human relationship (Cook et al., 2014, 2016, 2018; Karl, Boch, Zamansky, et al., 2020; Karl et al., 2021; and see e.g., Gábor et al., 2021; Berns et al., 2015 for investigations using other sensory modalities). The findings of these studies mainly reveal the involvement of subcortical structures. Although the present review focuses on neocortical structures, we briefly summarize these findings to understand how dogs perceive visual social cues related to their caregivers.

Overall, viewing their primary caregiver or handler compared to less or unfamiliar humans led to activation in limbic structures associated with emotion or reward processing, such as the amygdala, insula, caudate or cingulate cortex (Karl, Boch, Zamansky, et al., 2020; Thompkins et al., 2021). Findings so far also indicate a relationship between the quality of the dog-human relationship and the relative engagement of these areas (Cook et al., 2014, 2016, 2018; Karl, Boch, Zamansky, et al., 2020; Thompkins et al., 2021). For example, Thompkins et al. (2018) found a positive correlation between activation strength in limbic brain areas and the amount of time dogs spent looking at the familiar human handler when presented with an unsolvable task; Cook et al. (2018) showed that whether dogs choose to spend time with their primary caregiver vs. receiving food could be predicted based on activation in the reward-sensitive caudate nucleus (Berns et al., 2013) for a social (i.e., praise) vs. food reward. Two studies investigated the neural underpinnings of dogs observing their primary caregivers interacting with an unfamiliar real (Karl et al., 2021) or fake (Cook et al., 2018) dog to create a rivalry situation. Karl et al. (2021) found that observing a positive social interaction between their primary caregiver and another dog compared to an unfamiliar human elicited the greatest activation in the dog’s hypothalamus (Karl et al., 2021). Cook et al. (2018) found a positive correlation between amygdala activation and dog-directed aggression when dogs observed their primary caregiver feeding a fake dog, further evidencing the increased arousal of dogs in potential rivalry settings. Thus, investigations so far resulted in converging evidence that dogs respond to their primary human care-givers or handlers differentially than to other human agents and that the individual dog-human relationship quality, as well as the type of observed interactions, modulates these neural responses.

Overall, the reviewed dog neuroimaging findings suggest a pre-dominant role of the dog temporal lobe for social cognition, similar to humans and non-human primates (Braunsdorf et al., 2021). They also revealed agent- and action-responsive areas in the dog extrastriate and temporal cortex sensitive to invariant aspects of face and body perception, and further temporal cortex areas sensitive to dynamic aspects of visual social cues and action features. Action observation led to more pronounced temporal than parietal lobe engagement and elicited activation in somatosensory regions. These findings provide first evidence for partly analogous neural bases of agent and action perception in dogs and humans but also emphasize cross-species divergencies, with mixed evidence for face specialization in the dog temporal lobe and no involvement of parietal regions during action observation in dogs. Lastly, findings of differential neural responses towards their primary caregivers compared to other human agents, especially in limbic structures, align well with the close bond between dogs and humans.

### 2.4 Main challenges in the field and suggestions on how to overcome them

Awake dog neuroimaging, as outlined in this review, holds great promise for a better understanding of social dog brain function and related affect and cognition. However, as with any novel approach, there are several challenges to be overcome to reach the full potential of the methodology and to ultimately establish domestic dogs as a powerful comparative model species in comparative neuroimaging. We focus on two main challenges here.

#### 2.4.1 Insufficient data reporting and sharing

The first and most significant current limitation in the field is the need for more sufficient data reporting and sharing, making reviewing prior findings challenging. The field currently lacks an agreement for a shared template space analogous to the MNI space used extensively in human neuroimaging, or the NMT macaque (Seidlitz et al., 2018) and NIH marmoset (C. Liu et al., 2018) template spaces for primate neuroimaging. This might also be challenging to achieve considering the variation of the skull/brain shape and size across dog breeds (Bunford et al., 2017; Czeibert et al., 2020). However, multiple publicly available templates along with detailed anatomical atlases for the dog brain do exist, either based on structural scans of one specific breed (X. Liu et al., 2020) or single dog (Czeibert et al., 2019) or averaged across breeds (Datta et al., 2012; Johnson et al., 2020; Nitzsche et al., 2019). The majority of the studies conducting whole-brain analyses used a publicly available template and atlas (see Supplementary Table S1 for detailed information). Six studies did not report coordinates of activation peaks and often only scarcely described the results using somewhat vague anatomical descriptions, such as in which lobe they found activation (Cook et al., 2016; Cuaya et al., 2016; Dilks et al., 2015; Gillette et al., 2022; Phillips et al., 2022; Thompkins et al., 2018, 2021). In addition, none of the six studies made statistical maps of their group analysis publicly available, meaning one has to rely solely on the study figures to determine in which gyrus or sulcus area the authors found activation (which posed a major challenge for the present review). In general, only four (Boch, Karl, et al., 2023; Boch, Wagner, et al., 2023; Karl, Boch, Zamansky, et al., 2020; Karl et al., 2021) out of the sixteen studies deposited statistical maps and further study data open access on public repositories. Thus, considering the insufficient data reporting and lack of data sharing practices, gathering cumulative evidence is currently challenging and prevents quantitative meta-analyses (see e.g., Salimi--Khorshidi et al., 2009; Wager et al., 2007 for review of image- and coordinate-based approaches).

To overcome these limitations and set the foundation for researchers to build on each other’s work directly, the field of dog neuroimaging should adapt reporting standards from human neuroimaging and commit to reporting coordinates of activation peaks along with anatomical labels derived from publicly available templates. If in-house templates are used, they should be published with the study. These measures would enable researchers to convert coordinates between different template spaces, allowing for first coordinate-based meta-analyses. To achieve even more precise and statistically powered cumulative measures and in light of open science practices, group statistical maps of dog neuroimaging studies should be made publicly available, which will open up many new possibilities, such as more precise regions-of-interest (ROIs) beyond large anatomical masks of gyri or image-based meta-analyses.

#### 2.4.2 Statistical analysis - power, type I/II errors, and transparency

A unique aspect of dog neuroimaging is that the dogs are fully awake and unrestrained throughout training and data collection. Due to the intensive training regimes required to achieve this goal (Karl, Boch, Virányi, et al., 2020; Strassberg et al., 2019), dog fMRI studies typically have small sample sizes. For example, the studies reviewed here have a median sample size of 12 dogs (mean = 12.91, *SD* = 6.83, range: 2 – 28 dogs; see Table S1 for sample sizes). Thus, assuming similar effect sizes as in humans or other non-human animals, these studies might often have low statistical power to detect small to medium effect sizes. There are already numerous strategies from human neuroscience to deal with statistical power issues of studies with restricted samples (see e.g., Poldrack et al., 2017 for review); we will focus on the suggestions feasible for dog neuroimaging with a focus on the studies included in the review.

In terms of data analysis, statistical power could be increased by lowering the number of comparisons. This can be achieved by restricting investigations, for example, to *a priori* defined anatomical regions-of-interest (ROIs, see (Cook et al., 2014, 2018). Some studies have also successfully used independent functional localizer tasks or split the data set to determine search spaces, to have more restricted ROIs than anatomical masks (Boch, Karl, et al., 2023; Boch, Wagner, et al., 2023; Hernández-Pérez et al., 2018). However, to prevent questionable research questions, such as circular analysis (Kriegeskorte, Kyle Simmons, et al., 2009) and in the light of transparent and reproducible science, it is important to note that researchers should select ROIs before knowing the results and ideally preregister the hypotheses and analysis plan to provide a clear differentiation between exploratory and confirmatory analyses and results (none of the reviewed studies was preregistered).

As for any kind of empirical approach, researchers have to balance the risks of false negative and false positive results. Considering the limited sample sizes, another strategy to increase power, which comes at the cost of a higher false-positive rate though, is to use more liberal statistical thresholds (Boch, Wagner, et al., 2023; Bunford et al., 2020; Thompkins et al., 2018). This approach has also been suggested for human research areas dealing with limited participant numbers, such as clinical research (Poldrack et al., 2017). However, results have to be discussed more cautiously, and the reasoning for the lowered threshold should be transparently addressed and ideally preregistered beforehand.

It has also been shown that statistical power in dog neuroimaging could be significantly improved using a tailored dog haemodynamic response function (HRF) to analyse the data (Boch et al., 2021). However, despite the evidence that the dog BOLD signal peaks earlier in the dog visual cortex than expected based on the human HRF, most of the reviewed dog fMRI studies still use the latter (Bunford et al., 2020; Cook et al., 2014, 2016, 2018; Cuaya et al., 2016; Dilks et al., 2015; Gillette et al., 2022; Hernández-Pérez et al., 2018; Phillips et al., 2022; Szabó et al., 2020; Thompkins et al., 2018, 2021).

Regarding data collection, another approach, and also a common strategy in primate neuroimaging (Zanini et al., 2023), which has also been previously suggested for dog neuroimaging (Huber and Lamm, 2017), is to collect more extensive individual data. So far, most dog neuroimaging studies have a low number of individual trials (but see (Phillips et al., 2022), likely because not all dogs in training would be able to complete a higher number of individual task runs due to several reasons, such as the availability of the dog and primary caregiver, a high number of repetitions of scanning sessions due to too excessive motion, or lack of interest, habituation, and learning effect after (too) many repeats. However, based on our own experience, there are often exceptions in a dog sample, with some high-performing dogs achieving multiple successful data collection attempts in 1–2 data collection sessions (Boch, Wagner, et al., 2023). Thus, another way to increase statistical power could be to set a minimum task run requirement for a dog’s data to be included in the study sample (e.g., min. 2 task runs) but, if possible, to continue collecting more task runs within the planned data collection period.

Another avenue to improve signal detection in dog neuroimaging is the development of equipment optimized for dogs. The majority of the reviewed dog fMRI studies used coils developed for measurements of human body parts (Boch, Wagner, et al., 2023; Bunford et al., 2020; Cook et al., 2014, 2016, 2018; Cuaya et al., 2016; Dilks et al., 2015; Gillette et al., 2022; Hernández-Pérez et al., 2018; Karl, Boch, Zamansky, et al., 2020; Karl et al., 2021; Phillips et al., 2022; Szabó et al., 2020; Thompkins et al., 2018, 2021). Our research group recently developed the first coil array tailored for dog cranial and neuroanatomy and tests showed that the k9 (i.e., canine) head coil improves detection power and the signal-to-noise ratio of functional and structural measurements compared to the human knee coil (Guran et al., 2023). Our own observations (Karl et al., 2019) and the work of other research groups show that dogs of various dog breeds, including mixed-breeds, are able to successfully complete training to participate in awake dog neuroimaging. We, therefore, developed a coil to accommodate the varying head sizes and shapes with a manually adjustable chin rest in terms of height and position. This ensures the dog brain is always positioned as close as possible to the coil array. The neural bases of action observation in dogs have already been investigated using the k9 head coil (Boch, Karl, et al., 2023; voxel size 1.5 × 1.5 × 2 mm^3^). Dog neuroimaging researchers, including our own research group, are also working on the development of data acquisition sequences to improve detection power in dog frontal lobes. Based on our own tests, fieldmaps, which are typically used in human and non-human primate neuroimaging, cannot significantly improve measurements in frontal regions due to the severity of distortions and conducting studies with higher field strengths than the currently used 3 T may result in increased motion artefacts (Nowogrodzki, 2018). It thus seems that radically novel sequences and measurement approaches may be needed to overcome this major limitation in acquiring whole-brain measurements. Alternatives would be the use of imaging sequences targeted for measurements in the frontal lobes (e.g., change of phase encoding direction) or the application of other non-invasive imaging approaches, such as electroencephalography (see e.g., Boros et al., 2021; Törnqvist et al., 2013 for applications in dogs), which comes at the cost of less signal in other brain regions.

Lastly, as mentioned above, meta-analyses would also allow for higher-powered cumulative measures and provide more restricted ROIs, and sufficient study reporting would enable direct replications to test the reproducibility of prior findings. Increasing and more precise evidence about the location of previously identified areas would also allow the field to move on to more targeted a priori hypotheses about the specific roles of brain areas associated with the perception of visual social cues. Finally, another avenue to explore would be more collaborative efforts, such as neuroimaging editions of the ManyDogs project (ManyDogs Project et al., 2023).

Thus, while it may be difficult to increase sample sizes, numerous avenues are available to improve statistical power, which are hopefully increasingly applied in future investigations. Irrespective of these measures, we advocate for the pervasive use of open science practices, such as preregistration (if applicable; see Lakens, 2019), a clear declaration of confirmatory vs. exploratory analyses, and exhaustive open reporting of data and code. As in every scientific discipline, standards gradually evolve through critically examining prevailing practices. Human neuroimaging reporting and analysis standards also emerged from discussions surrounding, for example, multiple comparisons and inflated false-positive results (Bennett et al., 2009) circular analysis (Kriegeskorte, Simmons, et al., 2009; Vul and Pashler, 2012) or selection of ROIs after results are known (Poldrack et al., 2017). By reflecting on current limitations in dog neuroimaging and our own experiences and highlighting approaches that emerged from similar discourses in human neuroimaging research, we hope to stimulate a constructive discourse on how to improve existing standards within the field, thereby fostering its growth. Therefore, we are also strongly committed to enhancing our own data sharing and reporting standards.

### 2.5 Future directions

The dog fMRI studies investigating the neural bases of visual social cognition so far have localized cortical brain regions involved in face and body (i.e., agent) action perception and provide the foundation to ask more complex research questions and experimental designs.

Preliminary evidence is already suggestive of individual functions for some of the localized brain areas, such as perception of dynamic visual aspects of social cues in the sylvian gyrus (Boch, Karl, et al., 2023; Hernández-Pérez et al., 2018; Karl et al., 2021; Phillips et al., 2022) or sensitivity for species identity in the mid suprasylvian gyrus (Boch, Karl, et al., 2023; Bunford et al., 2020). However, more research is needed to uncover new layers of comparison between dog and human social cognition and identify the specific roles of each brain region (e.g., identity vs. emotion perception or motion vs. goal encoding). Studies could, for example, employ repetition suppression paradigms focusing on the identified brain areas to investigate functional specificity (see e. g., Gábor et al., 2020; Kilner et al., 2009 for applications in dogs and humans). Another outstanding research question is to investigate if the identified areas form connections analogous to the visual pathways identified in the human brain (Pitcher and Ungerleider, 2021; Ungerleider and Haxby, 1994; Wurm and Caramazza, 2022). Thus, more research is needed to understand how the identified social brain regions are embedded in the species’ brains. Combined investigations using task-based and resting-state neuroimaging data in primates have, for example, revealed important insights into the networks supporting face perception and social cognition (Roumazeilles et al., 2021; Schwiedrzik et al., 2015). Adopting this approach for dogs would not only allow for a better understanding of the neural bases supporting social cognition in dogs but also offer another level of comparison between humans and dogs.

Further, since a first understanding of the areas active when dogs see agents or actions is now established, investigations using more complex visual stimuli to study higher-order socio-cognitive skills can now be undertaken. Behavioural research has already shown that dogs have remarkable imitation skills (Fugazza et al., 2023; Huber et al., 2020; Range et al., 2007), that they form expectations about their physical and social environment (Lonardo et al., 2021; Schünemann et al., 2021; Virányi et al., 2006; Völter, Lonardo, et al., 2023; Völter, Tomašić, et al., 2023; Völter and Huber, 2021a, 2021b) or even perform visual perspective taking (Catala et al., 2017; Maginnity and Grace, 2014) (Huber and Lonardo, 2023), but the neural bases of these visual analogous socio-cognitive skills with humans still remain largely unstudied.

Importantly, to prevent reverse inference (Hutzler, 2014; Poldrack, 2006), it would be helpful to combine the neuroimaging data of passive viewing paradigms with behavioural measures. For reasons of task-related motion during data acquisition, it is challenging though to acquire behavioural data while underdoing functional MRI. However, depending on the research question, out-of-scanner behavioural or eye-tracking tasks, or questionnaires assessing, e.g., the dogs’ temperament, can provide important additional insights for the interpretation of the neuroimaging findings (Cook et al., 2016; Karl et al., 2020; Thompkins et al., 2021). Nonetheless, to prevent selective reporting, considering that many such behavioural measures and questionnaires can be collected, it is again important to note that analysis plans should ideally be preregistered or that explorative research should be transparently reported as such.

Finally, this review focuses on the neural bases of visual social cognition; while there is a growing body of evidence of shared visual social abilities with humans and primates, dogs of course also have highly sensitive olfactory and auditory senses. The neural bases of dog olfaction have not been investigated extensively yet (Berns et al., 2015; Jia et al., 2016; Ramaihgari et al., 2018), but dog neuroimaging studies already provide first comparative insights into how the dog brain processes social auditory cues (see e.g., Bálint et al., 2023; Gergely et al., 2023; or Andics and Miklósi, 2018 for review). We hope that overcoming current limitations in the field will allow for a better integration of all dog neuroimaging findings so far to learn more about how the dog brain processes and integrates multisensory social information.

## 3 Conclusion

Over the last decade, dog neuroimaging research has delivered first promising evidence of functional convergencies between the neural bases of dog and human visual social cognition. By building on this foundation and overcoming current challenges, dogs promise to become an even more powerful comparative model species, unravelling new insights into the evolutionary roots of social cognition.

## Supplementary Material

Supplementary Material
