# Supplementary material for: Domestic dogs as a comparative model for social neuroscience: Advances and challenges
Source: Neurosci Biobehav Rev. Author manuscript; Available in PMC 2024 Aug 7. (PMC7616343; doi:10.1016/j.neubiorev.2024.105700)
Supplement: Supplementary Material [file EMS196380-supplement-Supplementary_Material.pdf]

Supplementary Table S1. Overview of dog functional MRI studies investigating visual perception of social cues

study	main topics	dog *N*	human *N*	out-of-scanner	atlas	coordinates & labels	statmaps
Cook et al. (2014)	DHR	12	/	questionnaire	Datta	ROI	/
Dilks et al. (2015)	FBE	6	/	/	Datta	none	/
Cook et al. (2016)	DHR	15/13/15	/	behavioural task	Native space	ROI	/
Cuaya et al. (2016)	FBE	7	/	/	Datta	none	/
Cook et al. (2018)	DHR	13	/	questionnaire	Datta	ROI	/
Thompkins et al. (2018)	FBE	12*	/	/	in-house	none	/
Hernández-Pérez et al. (2018)	FBE	8/4	/	/	Datta	yes	/
Szabó et al. (2020)	FBE	13/11	/	/	Czeibert	yes	private repository
Karl et al. (2020b)	DHR	17	/	eye-tracking, behavioural task	Nitzsche / Czeibert	yes	yes
Bunford et al. (2020)	FBE	20	30	/	Czeibert	yes	/
Thompkins et al. (2021)	FBE / DHR	22/25*	/	behavioural task	in-house	no coordinates, anatomical labels	/
Karl et al. (2021)	DHR / ACT	12	/	/	Nitzsche / Czeibert	yes	yes
Phillips et al. (2022)	ACT	2	2	/	Johnson	no coordinates, one anatomical label	/
Gillette et al. (2022)	FBE	7	5	/	Datta	no coordinates, anatomical labels	/
Boch, Wagner, et al. (2023)	FBE	15	40	/	Nitzsche / Czeibert	yes	yes
Boch, Karl, et al. (2023)	ACT / FBE	28	40	/	Nitzsche / Czeibert	yes	yes

Note. Findings can be summarized into three main topics: FBE = face, body and emotion perception; ACT = action observation; DHR = dog-human relationship. Sample sizes (*N*) range from 2 to 28 dogs. The majority are pet dogs, others are in training to become detection dogs (marked with *). Five studies have comparative human samples; 5 DHR studies added out-of-scanner behavioural measures. The majority of studies used publicly available templates (Czeibert et al., 2019; Datta et al., 2012; Johnson et al., 2020; Nitzsche et al., 2019), two studies used an unpublished in-house template, one did not provide this information. Seven studies report peak coordinates along with anatomical labels retrieved from the respective templates ("yes"), three studies do not report peak coordinates but (some) anatomical labels, three studies only performed region-of-interest (ROI) analysis, and three studies do not report peak coordinates or anatomical labels beyond lobe descriptions. Four studies publicly share data including whole-brain group statistical maps (statmaps).
